# Knockdown of heterochromatin protein 1 binding protein 3 recapitulates phenotypic, cellular, and molecular features of aging

**DOI:** 10.1111/acel.12886

**Published:** 2018-12-13

**Authors:** Sarah M. Neuner, Shengyuan Ding, Catherine C. Kaczorowski

**Affiliations:** ^1^ The Jackson Laboratory Bar Harbor Maine; ^2^ Neuroscience Institute University of Tennessee Health Science Center Memphis Tennessee

**Keywords:** aging, cognitive decline, electrophysiology, transcriptomics, viral vectors

## Abstract

Identifying genetic factors that modify an individual's susceptibility to cognitive decline in aging is critical to understanding biological processes involved and mitigating risk associated with a number of age‐related disorders. Recently, heterochromatin protein 1 binding protein 3 (*Hp1bp3*) was identified as a mediator of cognitive aging. Here, we provide a mechanistic explanation for these findings and show that targeted knockdown of *Hp1bp3 *in the hippocampus by 50%–75% is sufficient to induce cognitive deficits and transcriptional changes reminiscent of those observed in aging and Alzheimer's disease brains. Specifically, neuroinflammatory‐related pathways become activated following *Hp1bp3 *knockdown in combination with a robust decrease in genes involved in synaptic activity and neuronal function. To test the hypothesis that *Hp1bp3 *mediates susceptibility to cognitive deficits via a role in neuronal excitability, we performed slice electrophysiology demonstrate transcriptional changes after *Hp1bp3 *knockdown manifest functionally as a reduction in hippocampal neuronal intrinsic excitability and synaptic plasticity. In addition, as *Hp1bp3 *is a known mediator of miRNA biogenesis, here we profile the miRNA transcriptome and identify mir‐223 as a putative regulator of a portion of observed mRNA changes, particularly those that are inflammatory‐related. In summary, work here identifies *Hp1bp3 *as a critical mediator of aging‐related changes at the phenotypic, cellular, and molecular level and will help inform the development of therapeutics designed to target either *Hp1bp3 *or its downstream effectors in order to promote cognitive longevity.

## 1 INTRODUCTION

Most individuals will experience some degree of cognitive decline during aging, although the extent of this decline can vary widely. As aging is the leading risk factor for many neurodegenerative diseases, including Alzheimer's, it is critical to understand why some individuals are at increased risk for age‐related cognitive decline and eventual dementia. Studies suggest up to 60%–70% of the variation observed in cognitive abilities during aging are attributable to genetic factors (McClearn et al., 1997), although precise variants involved have been difficult to identify. Recently, our laboratory identified heterochromatin protein 1 binding protein 3 (*Hp1bp3*) as a modifier of cognitive aging in mice (Neuner et al., 2016). Using a forward genetics screen in the BXD genetic reference panel, we identified a quantitative trait locus (QTL) on chromosome 4 which harbored variants responsible for variation in cognitive abilities at midlife. Using a combination of bioinformatics and functional validation approaches, *Hp1bp3 *emerged as a likely gene candidate responsible for mediating the QTL effect. Specifically, strains harboring the DBA2/J (D2) allele of *Hp1bp3 *exhibited decreased *Hp1bp3 *gene expression in the hippocampus in combination with exacerbated cognitive aging. This relationship between decreased *Hp1bp3 *and reduced cognitive function was observed in the *Hp1bp3 *knockout mouse as well as aging impaired humans (Neuner et al., 2016). However, the mechanistic link between *Hp1bp3 *and regulation of cognitive function remains to be elucidated. Therefore, the goal of this study was to better understand how *Hp1bp3 *influences cognitive function under baseline conditions in adult animals by using virally encoded shRNA targeting *Hp1bp3 *designed to reduce expression similar to that observed in aging impaired individuals.

*Hp1bp3 *is a heterochromatin binding protein that is related to the histone H1 family of proteins (Garfinkel, Melamed‐Book, Anuka, Bustin, & Orly, 2015). It has been implicated in a variety of functions, most notably selective regulation of gene expression via a role in the modulation of chromatin structure (Dutta et al., 2014) and micro‐RNA (miRNA) biogenesis in human cells (Liu et al., 2016). In our previous study, enrichment analysis of the hippocampal transcriptome in relation to *Hp1bp3 *identified negative correlates of *Hp1bp3 *significantly enriched for localization to the plasma membrane, with functional annotations including ion channel activity and G‐protein coupled receptor activity (Neuner et al., 2016). Plasma membrane ion channels and receptors are critical regulators of neuronal excitability and synaptic plasticity, leading candidate mechanisms for memory storage (Kandel, 2001). Taken together, this information led us to hypothesize that *Hp1bp3 *may be mediating its effects on cognitive longevity via the modulation of hippocampal neuronal excitability.

Synaptic plasticity mechanisms such as long‐term potentiation (LTP) have been identified as leading molecular and cellular mechanisms of memory storage based on their properties of that include input specificity and associativity (Bliss & Collingridge, 1993). Intrinsic neuronal excitability is intimately linked to these processes, as changes in the intrinsic excitability of a neuron will influence its ability to respond to input—ultimately influencing synaptic throughput and the ability of a neural network to undergo long‐lasting changes such as LTP (Zhang & Linden, 2003). Multiple lines of evidence support the contention that these mechanisms are critical for learning and memory. For example, aged cognitively impaired rodents exhibit significantly decreased intrinsic neuronal excitability relative to young or nonimpaired controls (Kaczorowski & Disterhoft, 2009), and pharmacological agents that block LTP have also been observed to impair cognitive function (Abraham & Mason, 1988).

De novo gene transcription is a fundamental biological process underlying LTP and corresponding modification of synapses. Upon neuronal activation, a highly specific cascade of gene transcription is initiated that, under the right conditions, results in stable and long‐lasting functional changes including synaptic localization of ion channels and receptors as well as growth and morphological alterations of both axons and dendrites (Gutierrez & Davies, 2011). As with LTP itself, multiple lines of evidence support the notion that gene transcription is critical for successful memory formation and consolidation, including studies that have demonstrated amnesic effects resulting from blocking mRNA synthesis (Da Silva et al., 2008). Multiple classes of genes have been implicated in plasticity‐related transcription, including transcription factors such as the cAMP‐response element‐binding protein (CREB) family and immediate early genes such as the activity‐regulated cytoskeleton‐associated protein (*Arc*) and *c‐Fos *(Alberini & Kandel, 2014).

However, it remains to be elucidated exactly how these transcriptional programs are activated and how variation in de novo gene transcription under baseline conditions may contribute to cognitive impairment. The precise nature of activity‐related gene transcription implicates modification of chromatin structure as critical for memory formation and consolidation, as specific genes need to be accessible by transcriptional machinery at defined intervals.

Here, we assess the effects of a hippocampus‐specific knockdown (KD) of *Hp1bp3 *on cognitive function, the hippocampal transcriptome, neuronal excitability, and synaptic plasticity. We perform these experiments in both the C57BL6/J (B6) and D2 inbred mouse strains to enhance the rigor of our approach, as recent findings suggest studies conducted in a single genetic background limit the generalizability of mouse studies (Sittig et al., 2016). We chose these two strains as they are the parental lines of the BXD genetic reference panel that was originally used to discover *Hp1bp3 *as a modifier of cognitive aging (Neuner et al., 2016; Peirce, Lu, Gu, Silver, & Williams, 2004). In addition, the B6 and D2 strains show marked phenotype differences in a variety of domains, including both cognitive function and hippocampal neuronal excitability (Matsuyama, Namgung, & Routtenberg, 1997; Nguyen, Abel, Kandel, & Bourtchouladze, 2000; Philip et al., 2010). It was our hypothesis that any effect of *Hp1bp3 *KD observed across strains would be robust and potentially translatable to diverse genetic contexts.

## 2 RESULTS

### 2.1 Hippocampus‐specific knockdown of *Hp1bp3* impairs cognitive function

**Figure 1:**
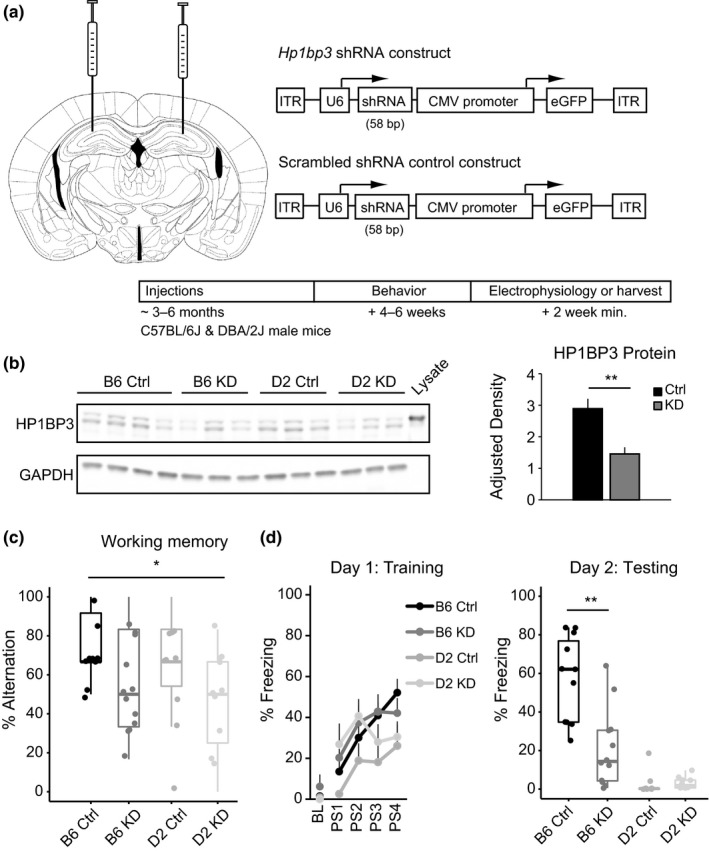
Hippocampus‐specific knockdown of *Hp1bp3* impairs cognitive function. (a) 1.0 μl of AAV9 encoding either *Hp1bp3 *shRNA or a scrambled control was injected bilaterally into the dorsal hippocampus of adult C57BL/6 J (B6) and DBA/2 J (D2) mice. After 4–6 weeks, a subset of mice were behaviorally tested. A minimum of two weeks later, mice were either harvested or used for electrophysiology. Mouse brain image adapted from The Mouse Brain in Stereotaxic Coordinates (Paxinos and Franklin, 2013). (b) Left, efficacy of the viral vector to reduce HP1BP3 levels in the hippocampus was confirmed via western blot; *n* = 6/group, two‐way ANOVA effect of strain *F*(1, 20) = 1.2 *p* = 0.3, effect of treatment *F*(1, 20) = 15.6, *p* = 0.001, no interaction. Right, data from four independent blots (two experiments) were pooled across strains (*n* = 12/group) and quantified. Lysate from human 293 T cells overexpressing HP1BP3 was used as a positive control. (c) Working memory was measured in the T‐maze. A significant effect of treatment was observed *F*(1, 41) = 5.9, *p* = 0.02, indicating that *Hp1bp3 *KD impairs working memory function across B6 and D2 mice. (d) Long‐term memory was assessed using contextual fear conditioning. Although B6 and D2 mice acquired the task differently on Day 1 [effect of strain, *F*(1, 39) = 5.4, *p* = 0.03], *Hp1bp3 *KD had no effect on the extent of these differences [effect of treatment, *F*(1, 39) = 0.12, *p* = 0.73]. Right, when mice were placed back in the training chamber on Day 2, D2 mice performed significantly worse than B6 mice [effect of strain *F*(1, 39) = 56.4, *p* < 0.001]—so poorly that further impairment caused by *Hp1bp3 *reduction could not be observed. However, *Hp1bp3 *KD did impair contextual fear memory in B6 mice [effect of treatment, *F*(1, 39) = 13.6, *p* = 0.001, interaction between strain and treatment *F*(1, 39) = 14.5, *p* < 0.001]. *main effect of treatment, *p* < 0.05, **t test, *p* < 0.05

In order to test the hypothesis that *Hp1bp3 *modifies cognitive function through a hippocampus‐specific effect rather than a peripheral effect (Garfinkel, Arad, et al., 2015), we designed an adeno‐associated virus serotype 9 (AAV9) viral vector encoding either shRNA for *Hp1bp3 *or a scrambled (scrmb) control and delivered 1.0 µl bilaterally to the dorsal hippocampus of adult (3–6 months) B6 and D2 mice (Figure 1a). Efficacy of the viral vector to reduce HP1BP3 levels was confirmed via western blot (Figure 1b). Notably, no reduction in HP1BP3 levels was observed in the cortex or cerebellum, indicating minimal viral spread outside our target region (Supporting Information Figure S1). Four to six weeks following injection, we assessed both working and long‐term contextual fear memory in these mice. There was a significant effect of *Hp1bp3 *KD on working memory as measured in the T‐maze, with both B6 and D2 mice receiving AAV9‐shRNA‐*Hp1bp3 *performing worse than their control counterparts [Figure 1b, two‐way ANOVA, effect of treatment *F*(1, 41) = 5.9, *p* = 0.02]. Long‐term contextual fear memory was then assessed via contextual fear conditioning. Although D2 mice overall performed more poorly than B6 mice during training, there was no effect of *Hp1bp3 *KD on contextual fear acquisition as measured by freezing during the 40 s following the final shock [Figure 1c, left, two‐way ANOVA, effect of strain *F*(1, 39) = 5.4, *p* = 0.03, effect of treatment *F*(1, 39) = 0.12, *p* = 0.73]. In contrast, there was a significant main effect of treatment on contextual fear memory [Figure 1c, right, two‐way ANOVA, effect of strain *F*(1, 39) = 56.4, *p* < 0.001, effect of treatment, *F*(1, 39) = 13.6, *p* = 0.001], suggesting *Hp1bp3 *is uniquely involved in mechanisms underlying memory consolidation and/or recall, but not fear acquisition. This effect was driven by the B6 strain, as the D2 mice performed so poorly no additional effect of treatment was observed, as evidenced by a significant interaction between strain and treatment [interaction between strain and treatment *F*(1, 39) = 14.5, *p* < 0.001]. These effects were not due to nonspecific effects on anxiety, activity, or overall growth as measured by open‐arm entries on the elevated plus maze, total arm entries, speed, and weight, respectively (Supporting Information Figure S2).

### 2.2 *Hp1bp3* knockdown alters specific aspects of the hippocampal transcriptome

**Figure 2:**
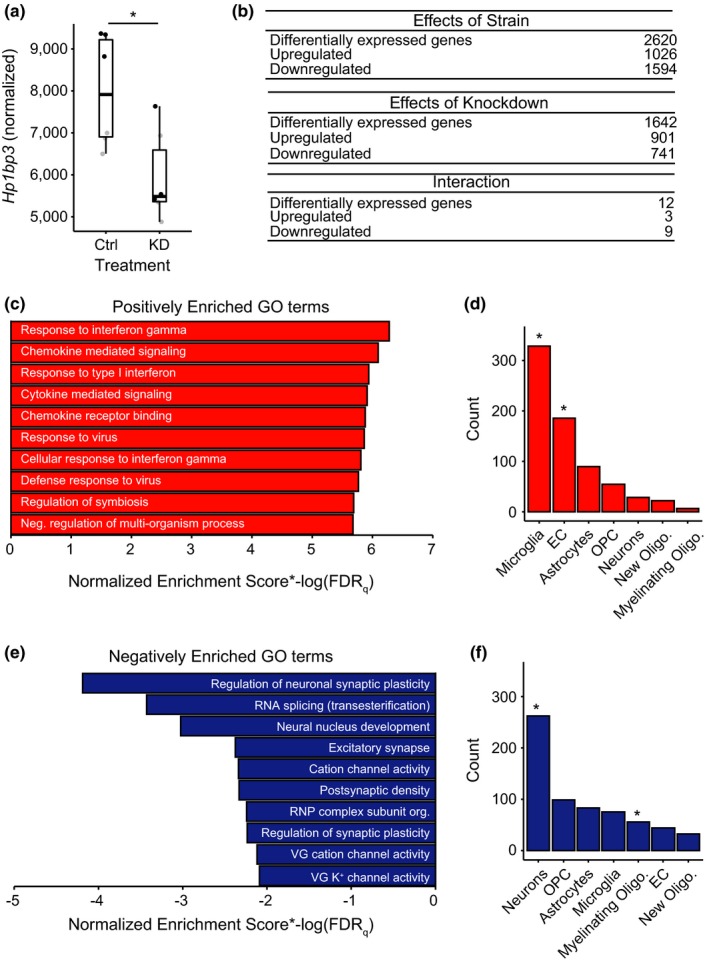
Knockdown of *Hp1bp3 *alters specific aspects of the hippocampal transcriptome. (a) Knockdown of *Hp1bp3 *was confirmed at the mRNA level [two‐way ANOVA, effect of strain *F*(1, 8) = 2.6, *p* = 0.02, effect of treatment *F*(1,8) = 17.1, *p* = 0.003, no interaction between strain and treatment]. *main effect of treatment, p < 0.05. (b) *Hp1bp3 *knockdown largely effects B6 and D2 mice similarly. Effect of strain: Upregulated genes are more highly expressed in B6 hippocampus. Effect of treatment: Upregulated genes are more highly expressed in *Hp1bp3 *KD hippocampus. (c) Among genes upregulated by *Hp1bp3 *KD that display a cell type specificity in their expression, there was a significant enrichment for immune‐related gene ontology (GO) terms. (d) There was a significant overlap between genes upregulated by *Hp1bp3* KD and genes most highly expressed in endothelial cells (EC) and microglia (hypergeometric test, *p* < 0.001). *, *p* < 0.05. (e) Among genes downregulated by *Hp1bp3 *KD, there was a significant enrichment for terms related to neuronal excitability, structure, and function. (f) There was a significant overlap between genes downregulated by *Hp1bp3 *KD that also display a cell type specificity in their expression and genes most highly expressed in neurons and myelinating oligodendrocytes (hypergeometric test, *p* < 0.001). *, *p* < 0.05

We next wanted to determine the mechanism(s) by which hippocampal KD of *Hp1bp3 *may be impairing cognitive function. We performed total RNA sequencing on whole hippocampal lysates from three mice per strain/treatment group and confirmed knockdown of *Hp1bp3 *at the mRNA level (Figure 2a). A subset of genes were identified to as differentially expressed relative to both strain background and treatment group, while relatively few genes displayed a significant strain by treatment interaction, suggesting *Hp1bp3 *KD alters the hippocampal transcriptome comparably across background strain (Figure 2b and Supporting Information Table S1). To identify specific pathways and functional categories altered by *Hp1bp3 *KD, we next utilized gene set enrichment analysis (GSEA) in order to identify gene ontology (GO) terms significantly enriched among differentially expressed genes. Among genes upregulated by *Hp1bp3 *KD, we identified a significant enrichment for immune‐related terms, including response to interferon gamma, chemokine‐mediated signaling, and response to type I interferon (Figure 2c). This increase in inflammatory signaling seemed to be driven largely by an increase in microglia, as genes upregulated by *Hp1bp3 *KD that also display a significant cell type‐specific expression (Zhang et al., 2014) showed a significant overlap with a list of genes most highly expressed by microglia (hypergeometric test *p* < 0.001, Figure 2d). In contrast, among genes downregulated by *Hp1bp3 *KD, we identified a significant enrichment for a number of terms related to neuron structure and function, including regulation of neuronal synaptic plasticity, excitatory synapse, and various channel activity terms (Figure 2e). In addition, among genes downregulated by *Hp1bp3 *KD, there was a significant enrichment for transcripts experimentally observed to be most highly expressed in neurons and myelinating oligodendrocytes (hypergeometric test, *p* < 0.001).

**Figure 3:**
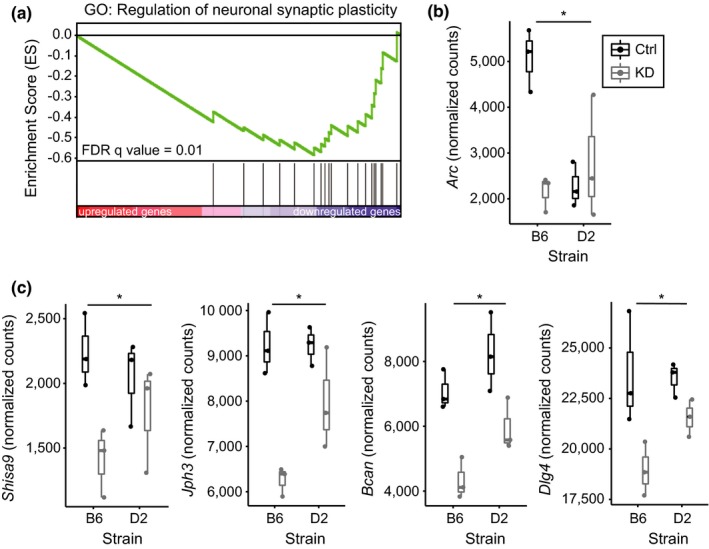
*Hp1bp3* KD negatively regulates neuronal synaptic plasticity. (a) Enrichment plot illustrating enrichment of the GO term regulation of neuronal synaptic plasticity among genes downregulated by *Hp1bp3 *KD. Enrichment score = −0.6, Normalized enrichment score = −2.2, FDR = 0.01. (b) The core driver gene most significantly downregulated by *Hp1bp3 *KD, particularly in B6 mice, was *Arc *(DESeq2 effect of treatment; log2FC = −1.2, adjusted *p* = 0.001, effect of strain; log2FC = −1.2, adjusted *p* = 0.001, interaction adjusted *p* = 0.2). (c) A number of plasma membrane proteins, particularly ion channels and receptors, were identified as additional core drivers of this enrichment. *DESeq2 main effect of treatment, *p* < 0.05

As synaptic plasticity is a leading candidate mechanism for information and memory storage, and due to the fact that we identified regulation of ion channels and receptors as a candidate mechanism for *Hp1bp3*‐induced cognitive impairment in an aging mouse population (Neuner et al., 2016), we were particularly interested in the most significantly downregulated GO term, regulation of neuronal synaptic plasticity. The identification of this pathway was driven by 19 genes strongly downregulated by *Hp1bp3 *KD (normalized enrichment score = −2.2, FDR = 0.01, Figure 3a). Of these 19 genes, 14 were identified as core driver genes by GSEA. Notably, the core driver gene most significantly downregulated by treatment, particularly in B6 mice, was the activity‐regulated cytoskeleton‐associated protein (*Arc*), a gene repeatedly implicated in learning and memory but for whom the upstream regulators remain poorly defined [(Shepherd & Bear, 2011), Figure 3b]. Additional core driver genes included a number of ion channels and receptors, including: (a) shisa family member 9 (*Shisa9*), an AMPAR auxiliary subunit (von Engelhardt et al., 2010), (b) junctophilin 3 (*Jph3*), a transmembrane junctional protein implicated in the regulation of the slow after‐hyperpolarization (Moriguchi et al., 2006), (c) brevican (*Bcan*), an extracellular matrix protein that regulates the speed of synaptic transmission (Blosa et al., 2015), and (d) disks large homolog 4 (*Dlg4*), also known as postsynaptic density protein 96 (*Psd95*), which plays a critical role in organizing postsynaptic signaling (Chen et al., 2011).

### 2.3 *Hp1bp3* knockdown decreases intrinsic excitability and synaptic plasticity of hippocampal neurons

**Figure 4:**
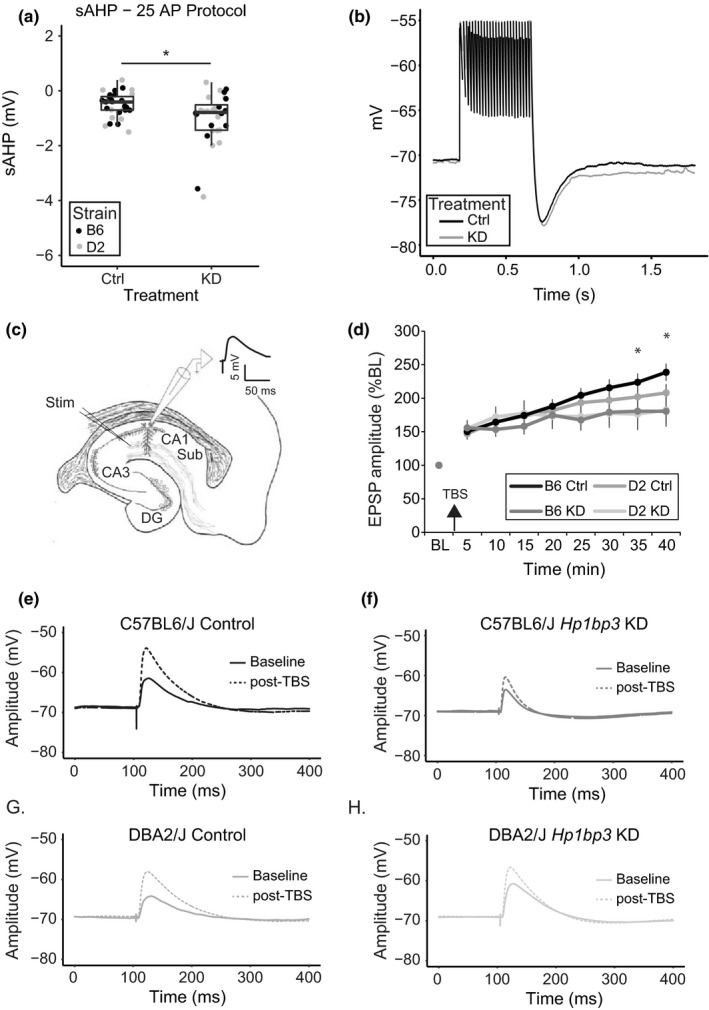
*Hp1bp3 *KD decreases intrinsic neuronal excitability and synaptic plasticity. (a) 25 action potentials were trigged by somatic current injection, and the slow after‐hyperpolarization was recorded 1 s after the last stimulus offset. sAHPs were not different across B6 and D2 mice [effect of strain, *F*(1,56) = 0.1, *p* = 0.7] so data presented are pooled across strains. A significant effect of treatment was detected [*F*(1, 56) = 6.7, *p* = 0.01], indicating that across strains, *Hp1bp3 *increases the sAHP. (b) Representative traces from Ctrl and KD animals. (c) Diagram of recording configuration for synaptic experiments. A stimulating electrode was placed in the Shaffer collateral leading from CA3 to CA1. Single neurons were recorded from in CA1 using whole‐cell patch‐clamp electrophysiology. (d) Excitatory postsynaptic potentials (EPSPs) were evoked once every 20 s for five minutes to obtain a stable baseline measure (BL). Theta‐burst stimulation (TBS) was delivered and EPSPs continued to be evoked and measured every 20 s for 35 min. Data were pooled into five‐minute bins for further analysis. A significant within‐subjects effect of time was observed [two‐way repeated measures ANOVA, Greenhouse‐Geisser corrected results, *F*(2, 155) = 18.3, *p* < 0.001], as EPSP amplitude increased drastically after TBS. A significant within‐subjects interaction between time and treatment was observed [*F*(2, 185) = 4.2, *p* = 0.01], and further posthoc testing indicated that control and *Hp1bp3 *KD groups were significantly different at the two latest time points tested (*p < 0.05). No interaction between time and strain was observed [*F*(2, 185) = 1.0, *p* = 0.4], indicating that *Hp1bp3 *knockdown impaired LTP similarly across strains. (e) Representative traces from B6 Ctrl, (f) B6 KD, (g) D2 Ctrl, and (h) D2 KD animals both pre‐ and post‐TBS

To directly test the validity of the findings that *Hp1bp3 *KD alters neuronal excitability and synaptic plasticity, we performed slice electrophysiology to measure the properties of neurons from either mice receiving AAV9‐shRNA‐*Hp1bp3 *or AAV9‐scrmb‐shRNA. Specifically, mice were sacrificed at least 6 weeks following viral injection and brains quickly removed, sectioned, and maintained in aCSF for recordings. For the subset of mice that were behaviorally tested, slice electrophysiology occurred at least two weeks after the conclusion of behavioral testing to allow hippocampal neurons to return to baseline conditions. We first examined intrinsic neuronal properties and found *Hp1bp3 *KD significantly increased the slow after‐hyperpolarization (sAHP) of hippocampal pyramidal neurons [Figure 4, two‐way ANOVA, effect of treatment *F*(1,56) = 6.7, *p* = 0.01, no main effect of strain]. This seemed to be a relatively specific effect, as resting properties such as resting membrane potential and input resistance of the cell were not altered by knockdown, although strain‐specific differences were observed (Supporting Information Figure S3). In addition, *Hp1bp3 *KD did not change the intrinsic firing properties of the neurons, as measured by threshold required to fire an action potential and the peak after‐hyperpolarization (Table 1).

**Table 1:** Basic intrinsic excitability characteristics across groups

Group	RMP (mV)	IR (MΩ)	AP_threshold_ (mV)	AHP_peak_ (mV)
B6 Ctrl	−64.3 ± 0.5	154.8 ± 8.7	−50.0 ± 0.7	−4.2 ± 0.6
B6 KD	−64.0 ± 0.5	135.0 ± 10.0	−50.5 ± 1.0	−4.2 ± 0.5
D2 Ctrl	−62.3 ± 0.5	192.9 ± 15.6	−48.6 ± 0.6	−5.1 ± 0.6
D2 KD	−61.3 ± 0.2	188.8 ± 0.9	−48.9 ± 0.8	−4.6 ± 0.5

Note

AHP: after hyperpolarization; AP: action potential; Ctrl: control; IR: input resistance; KD: knockdown; mV: millivolts; MΩ: megaohms; RMP: resting membrane potential.

In a separate cohort of mice, we next assessed the role of *Hp1bp3 *in synaptic plasticity by placing a stimulating electrode in the Shaffer collateral pathway and eliciting excitatory postsynaptic potentials (EPSPs) in CA1, measured via whole‐cell recordings (Figure 4c). Stimulation was titrated so that EPSPs 5 mV in amplitude were elicited once every 20 s for 5 min to obtain a stable baseline EPSP measurement. Theta‐burst stimulation (TBS), a paradigm shown to elicit long‐term potentiation in the Shaffer collateral (Graves, Moore, Spruston, Tryba, & Kaczorowski, 2016), was delivered immediately following five minutes of baseline recording. EPSPs continued to be elicited once every 20 s for 35 min to measure the change in EPSP amplitude post‐TBS. Data were collected and pooled into 5‐min bins for further analysis (Figure 4d). Mice receiving AAV9‐*Hp1bp3*‐shRNA displayed significantly impaired LTP relative to mice receiving AAV9‐scrmb‐shRNA, particularly at late time points [Figure 4e‐H]. No significant effects of strain, or interaction between time and strain, were observed, indicating that *Hp1bp3 *KD impaired synaptic plasticity similarly across groups. Notably, *Hp1bp3 *KD did not impair baseline characteristics such as intrinsic excitability as measured by the number of action potentials fired in response to a 1‐s sustained current injection (Supporting Information Figure S4a), amplitude of EPSPs in response to increasing stimulation intensities (Supporting Information Figure S4b), or paired pulse ratio (Supporting Information Figure S4c). In summary, these data demonstrate *Hp1bp3 *specifically regulates neuronal excitability and plasticity necessary for successful cognitive performance on both working and contextual fear memory tasks.

### 2.4 *Hp1bp3* selectively regulates a subset of micro‐RNAs

**Figure 5:**
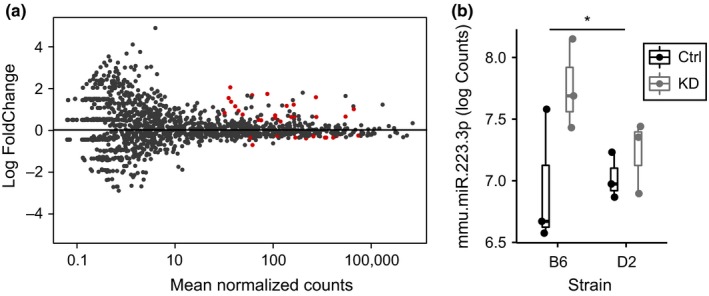
*Hp1bp3 *selectively regulates the miRNA transcriptome in vivo. (a) Plot illustrating (*x*‐axis) average miRNA expression versus (*y*‐axis) log fold change as measured by DESeq2. Red dots illustrate those miRNA transcripts that were determined to be differentially expressed relative to treated at an adjusted *p* = 0.10. (b) miRNA mmu‐mir‐223‐3p was identified as a putative upstream regulator of observed mRNA changes after *Hp1bp3 *KD and was predicted to be activated in our dataset by IPA. mmu‐mir‐223‐3p was significantly upregulated by *Hp1bp3 *knockdown [*two‐way ANOVA, effect of treatment *F*(1, 8) = 5.6, *p* = 0.046, no significant effect of strain or interaction between strain and treatment]

Recent studies have demonstrated that HP1BP3 plays a role in miRNA biogenesis in human cells in vitro (Liu et al., 2016). To investigate whether observed transcriptional changes were due to a global change in miRNA biogenesis, we analyzed the small RNA sequences generated by our initial total RNA sequencing on hippocampal tissue from 3 mice/group. Of 1915 miRNAs identified and tested for differential expression analysis, only 35 showed differential expression relative to treatment at an adjusted *p*‐value of 0.10 (Figure 5a and Supporting Information Table S2), with only 9 remaining significant at an adjusted *p* = 0.05. Thus, <0.5% of all miRNAs tested exhibit significant differential expression following *Hp1bp3 *KD. This is in comparison with mRNA, where just over 7% of all mRNAs tested were differentially expressed relative to *Hp1bp3 *KD (proportions of differentially expressed genes significantly different, *Z* = 11.5, *p* < 0.001), suggesting *Hp1bp3 *plays a much broader role in the regulation of mRNA expression over miRNA expression. These results demonstrate that in contrast to what has been observed in human cells, *Hp1bp3 *does not seem to be a global regulator of miRNA biogenesis in vivo in the mouse hippocampus.

Although global changes in miRNA expression were not observed, we next tested the hypothesis that the select miRNAs regulated by *Hp1bp3 *may explain downstream observed changes in mRNA expression and synaptic function. First, we input the list of mRNAs differentially expressed relative to treatment into Ingenuity Pathway Analysis (IPA) software (Kramer, Green, Pollard, & Tugendreich, 2014) and used the Core Analysis function to identify putative miRNA upstream regulators of our observed mRNA changes (Supporting Information Table S3). A number of miRNAs were identified as putative upstream regulators, as measured by significant overlap of known target genes and genes present in our list of differentially expressed mRNA. The top predicted upstream regulator, mir‐21, was predicted to be inhibited in our dataset based on the direction of change in its target molecules. However, the pattern of mir‐21 differential expression after *Hp1bp3 *KD did not fit this pattern, eliminating mir‐21 as a miRNA that could possible explain mRNA changes observed in our hands (Supporting Information Table S3). Next, we examined the miRNA identified as the second most likely miRNA upstream regulator, mir‐223. Based on the direction of change of its target molecules, IPA predicted that mir‐223 was activated in our dataset, and indeed, mir‐223 (specifically mmu‐mir‐223‐3p) was observed to be upregulated after *Hp1bp3 *KD (two‐way ANOVA, effect of treatment *F*(1, 8) = 5.6, *p* = 0.046, Figure 5b) and was one of the miRNA transcripts identified as significantly differentially expressed by DESeq2 at an adjusted *p* = 0.10 (mmu‐mir‐223‐3p adjusted *p* = 0.09, log2FC = 1.2). mir‐223, best studied in the context of hematopoietic stem cells, has been shown to function as a key modulator in the differentiation and activation of myeloid cells and has been implicated in a number of immune system functions (Yuan et al., 2018). In accordance with these known functions, the list of mir‐223 targets differentially expressed after *Hp1bp3 *KD was identified to be most highly enriched for the GO Biological Process term “immune system process” (FDR < 0.001) through WebGestalt over‐representation enrichment analysis (Wang, Duncan, Shi, & Zhang, 2013). Notably, mir‐223 has been shown to promote maturation, proliferation, and activation of myeloid cells (Tsitsiou & Lindsay, 2009), which overlaps with our previous observation that the upregulation of immune‐related genes following *Hp1bp3 *KD is likely due to an increase in numbers of microglia (Figure 2d).

### 2.5 Transcriptome‐level changes induced by *Hp1bp3* knockdown recapitulate those observed in human aging

**Figure 6:**
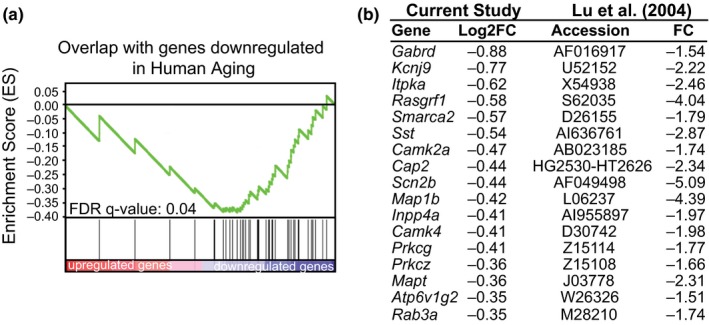
Transcriptome‐level changes induced by *Hp1bp3 *knockdown recapitulate those observed in human aging. (a) Enrichment plot illustrating overlap of genes downregulated in the human aging cortex with genes among genes downregulated by *Hp1bp3 *KD. Enrichment score = −0.4, Normalized enrichment score = −1.9, FDR = 0.04. (b) List of twenty genes downregulated by *Hp1bp3 *KD and their corresponding fold changes in the human aging cortex as reported by Lu et al. (2004)

In addition to standard GO terms and functional pathways, GSEA also allows gene lists of interest to be compared against experimentally derived gene sets that have been uploaded into the Molecular Signatures Database (Liberzon et al., 2011). When we compared our list of differentially expressed mRNAs to the chemical and genetic perturbations (CPG) database, we observed significant overlap between genes downregulated by *Hp1bp3 *KD and those genes downregulated with age in the human frontal cortex [(Lu et al., 2004), FDR *q*‐value = 0.04, Figure 6a]. This significant enrichment was driven by 43 genes significantly downregulated in both the aging human frontal cortex and the mouse hippocampus following *Hp1bp3 *KD, 37 of which were determined by GSEA to be core drivers of enrichment. The twenty most significantly downregulated core drivers, as well as their corresponding fold changes from the human brain (Lu et al., 2004), are listed in Figure 6b. As a group, this set of commonly downregulated genes displayed relatively diverse enrichment for GO Biological Process terms using WebGestalt over‐representation enrichment analysis (Wang et al., 2013), including interleukin secretion (IL‐5 FDR = 0.03, IL‐13 FDR = 0.03), microtubule polymerization (FDR = 0.04), and chemical synaptic transmission (FDR = 0.04), all processes known to be altered in aging. Combined with our previous observation that HP1BP3 expression is decreased in cognitively impaired aging humans, the overlap of the broader *Hp1bp3 *KD transcriptome with that of human aging supports the idea that *Hp1bp3 *is a critical regulator of a number of the transcriptional changes that occur during aging and may serve as a valuable therapeutic target to delay or prevent aging‐related cognitive decline.

## 3 DISCUSSION

### 3.1 Hippocampal *Hp1bp3* regulates cognitive function, gene transcription, and synaptic plasticity

Here, we demonstrate for the first time that a targeted knockdown of *Hp1bp3 *in the hippocampus by 50%–75% was sufficient to recapitulate cognitive deficits observed previously in global *Hp1bp3 *knockout mice (Neuner et al., 2016).

Specifically, in both cases, we observed impairments in both spatial working memory and contextual fear memory, but not in contextual fear acquisition. This implicates a relatively specific role for *Hp1bp3 *in mechanisms underlying working memory as well as memory consolidation and recall, but highlights that contextual fear acquisition may rely on independent mechanisms for successful completion. Overall, results here demonstrate that cognitive deficits caused by *Hp1bp3 *deficiency, presumably in both KO mice and aged impaired mice, are due primarily to *Hp1bp3 *deficiency in the hippocampus. This is an important finding, as *Hp1bp3 *knockout mice are smaller than WT littermates and have alterations in insulin signaling that may have impacted cognitive performance due to peripheral mechanisms (Garfinkel, Arad, et al., 2015). In addition, we extend our previous finding that loss of *Hp1bp3 *has a negative impact in cognitive function and provide mechanistic insight into how these alterations occur. Specifically, we show *Hp1bp3 *KD negatively regulates a number of genes critical for synaptic plasticity and neuronal activity, including the immediate early gene *Arc *(Figure 2b). Although *Arc *is well‐known to be critical for memory formation (Guzowski et al., 2000; Plath et al., 2006), the precise signaling cascades involved in *Arc *transcription and regulation are not well‐defined (Shepherd & Bear, 2011).

Here, we discover a novel role for *Hp1bp3 *as a regulator of *Arc *expression, which implicates *Hp1bp3 *in the broader context as a master regulator of transcriptional network changes required for successful induction of synaptic plasticity, likely via its role in modifying chromatin structure (Dutta et al., 2014). Finally, we demonstrate that observed transcriptional changes functionally manifest as impairments in both intrinsic neuronal excitability and synaptic plasticity. Together, these results provide important insight into physiological functions of *Hp1bp3 *under baseline conditions in adult mice. As a result, we can now better understand how misregulation of *Hp1bp3 *may contribute to cognitive impairments seen in both *Hp1bp3 *knockout mice and cognitively impaired aging mice and humans with decreased levels of *Hp1bp3 *(Neuner et al., 2016).

### 3.2 *Hp1bp3* effect on cognition and neural function is robust to genetic context

It has long been known that genetic background is critical for modifying phenotypic presentation. Recently, this was highlighted by a survey of 30 inbred lines, where in some cases, opposite conclusions were drawn regarding the effect of gene knockout depending on the genetic context of the manipulation (Sittig et al., 2016). Therefore, we thought it important to evaluate the effect of *Hp1bp3 *manipulation in more than one genetic background. We performed our experiments in the B6 and D2 inbred strains, as these strains were used to derive the BXD genetic reference panel which was originally used for identifying *Hp1bp3 *as a modifier of cognitive aging. These two strains show marked differences in learning and memory abilities (Neuner et al., 2016; Philip et al., 2010) as well as differences in certain types of LTP (Matsuyama et al., 1997; Nguyen et al., 2000). In general, our data are consistent with this literature, with the B6 strain outperforming the D2 strain on contextual fear conditioning. While we demonstrate here TBS is capable of inducing LTP in both strains, other types of LTP have been shown to be reduced in the D2 strain (Schimanski & Nguyen, 2005), which may explain their poor performance on contextual fear conditioning—a task which requires synaptic plasticity (Tang et al., 1999). Poor performance on contextual fear conditioning may also be explained, in part, by lower baseline levels of *Hp1bp3 *mRNA observed in the D2 strain (Figure 2a), which while not detected at the protein level (Figure 1b), is consistent with our previous findings that BXD strains carrying the *D *allele of *Hp1bp3 *show decrease hippocampal *Hp1bp3 *expression. As *Hp1pb3 *knockdown impaired cognitive function and reduced neuronal excitability in both strains, as well as largely affected the transcriptome across strains similarly (Figure 2b), our data suggest *Hp1bp3 *deficiency has a robust detrimental effect on cognitive function regardless of genetic context. This broadens the translational relevance of this finding, as restoring *Hp1bp3 *levels (or supplementing with a small molecule that increases *Hp1bp3 *levels [e.g., Metformin (Neuner et al., 2016)]), is likely to be beneficial to a wider variety of subjects than a target with context‐dependent effects.

### 3.3 *Hp1bp3* as a regulator of miRNA biogenesis

Contrary to previous reports (Liu et al., 2016), *Hp1bp3 *does not seem to be a global regulator of miRNA biogenesis, at least in the adult mouse hippocampus. Multiple explanations for these conflicting results exist, including different experimental species (mouse vs. human), differences in experimental techniques (in vitro vs. in vivo), and even differences in miRNA alignment protocols. In addition, we did observe a select set of miRNAs differentially expressed relative to *Hp1bp3 *KD, suggesting *Hp1bp3 *does regulate biogenesis of at least some miRNAs in vivo. Of particular interest to this study is mir‐223, which was identified by IPA as a putative upstream regulator of observed mRNA‐level changes following *Hp1bp3 *KD and whose expression in our miRNA sequencing data changed concordantly with that predicted by IPA. As a number of immune‐related genes have been shown to be downregulated in mir‐223 deficient cells (Lu et al., 2013), the large number of immune‐related genes upregulated after *Hp1bp3 *KD may, in part, be due to observed upregulation of mmu‐mir‐223‐3p. While mir‐223 is best studied in the context of the hematopoietic system, recent studies have investigated its role in CNS functions and it has recently been shown to be enriched in the hippocampus relative to other brain regions (Harraz, Eacker, Wang, Dawson, & Dawson, 2012). In particular, mir‐223 has been shown to decrease total dendritic tree length, branch number, and complexity in cell culture (Harraz, Xu, Guiberson, Dawson, & Dawson, 2014) and specifically target glutamate receptor transcripts for degradation in vivo (Harraz et al., 2012), which may link mir‐223 to some of the additional neuronal phenotypes observed in *Hp1bp3 *KD mice. In summary, while miRNA biogenesis likely contributes to some of the transcriptional changes observed after *Hp1bp3 *KD, additional mechanisms regulating gene expression are likely at play, including alterations in chromatin structure that result in changes in the accessibility of key genomic regions to necessary transcriptional machinery.

### 3.4 *Hp1pb3* knockdown recapitulates symptoms of aging and Alzheimer's disease

Many alterations observed after *Hp1bp3 *KD appear to phenocopy alterations observed in aging and Alzheimer's disease (AD). In particular, studies have identified decreases in neuronal excitability as cellular correlates of cognitive impairments in aging animals (Disterhoft, Wu, & Ohno, 2004). Notably, the sAHP is increased in aged impaired animals relative to young and aged nonimpaired animals (Kaczorowski & Disterhoft, 2009). Similar neuronal phenotypes have been observed in animal models of AD (Kaczorowski, Sametsky, Shah, Vassar, & Disterhoft, 2011). As these aged and AD neurons are less able to respond adequately to input, this reduced neuronal excitability translates into a reduction in the maintenance of LTP and poorer memory storage. Transcriptionally, mRNA changes after *Hp1bp3 *KD significantly overlap mRNA changes observed in the aging human cortex [FDR < 0.05, Figure 6, (Lu et al., 2004)] and are reminiscent of increased inflammation and neurodegeneration observed in AD patients (Van Eldik et al., 2016). In addition, baseline reductions in *Arc *expression have been observed in aging rodents, which correlates with less efficient memory storage and retrieval during aging (Penner et al., 2011). Each of these hallmark features of aging and AD—decreased neuronal excitability, reduced synaptic plasticity, impaired cognitive function, and increased neuroinflammation—is recapitulated in our *Hp1bp3 *KD animals. Together with our previous observations that cognitively impaired mice and humans have naturally occurring reductions in *Hp1bp3 *levels, our data suggest reduced *Hp1bp3 *is a relevant driver of aging and AD‐related phenotypes. As aging is the leading risk factor for many disease in addition to AD, and there is currently no cure for AD, understanding the origin of these deficits is critical for developing effect therapeutics to maintain healthspan in aging individuals. The identification and characterization of *Hp1bp3*’s effect on cognition and neural function under baseline adult conditions are a critical first step toward understanding how aging, *Hp1bp3*, and additional genetic and environmental factors interact to modify an individual's susceptibility to age‐related cognitive decline.

## 4 CONCLUSIONS AND FUTURE DIRECTIONS

Overall, our data support a critical role for *Hp1bp3 *in modifying cognition and neuronal function, as well as the transcriptional programs underlying both. These results further contribute to our understanding of both physiological functions of *Hp1bp3 *and molecular mediators of memory formation and storage, as well as some of the hallmark symptoms of aging. In addition to the selective regulation of gene expression, previous studies have implicated *Hp1bp3 *in the regulation of chromatin structure (Dutta et al., 2014). Recent studies have demonstrated that long‐term maintenance of chromatin structure is critical for maintenance of cognitive longevity (Lopez‐Otin, Blasco, Partridge, Serrano, & Kroemer, 2013), providing an additional mechanism *Hp1bp3 *may work through in order to influence cognitive aging. Future studies will address this possibility directly with targeted ATAC‐seq (an assay for transposase accessible chromatin with high‐throughput sequencing) in order to assess how chromatin accessibility changes with *Hp1bp3 *KD. The discoveries made here, as well as in future studies, will help inform the development of therapeutics designed to target either *Hp1bp3 *or its downstream effectors (such as those plasma membrane ion channels and receptors highlighted in Figure 3c) in order to help promote cognitive longevity and reduce the risk of multiple age‐related diseases.

## CONFLICT OF INTEREST

The authors declare no conflicts of interest.

## AUTHOR CONTRIBUTIONS

SMN, SD, and CCK conceived of, and designed, the experiments. SMN conducted the surgical and behavioral experiments as well as the mRNA and miRNA analyses, while SD conducted the electrophysiological experiments. SMN, SD, and CCK analyzed data and interpreted results. SMN and CCK wrote the manuscript, and all authors reviewed and approved the final manuscript.
