# Supplementary material for: Knockdown of heterochromatin protein 1 binding protein 3 recapitulates phenotypic, cellular, and molecular features of aging
Source: Aging Cell. 2018 Dec 13;18(1):e12886. doi: 10.1111/acel.12886 (PMC6351847; doi:10.1111/acel.12886)
Supplement: Supplementary file 1 [file ACEL-18-e12886-s001.pdf]

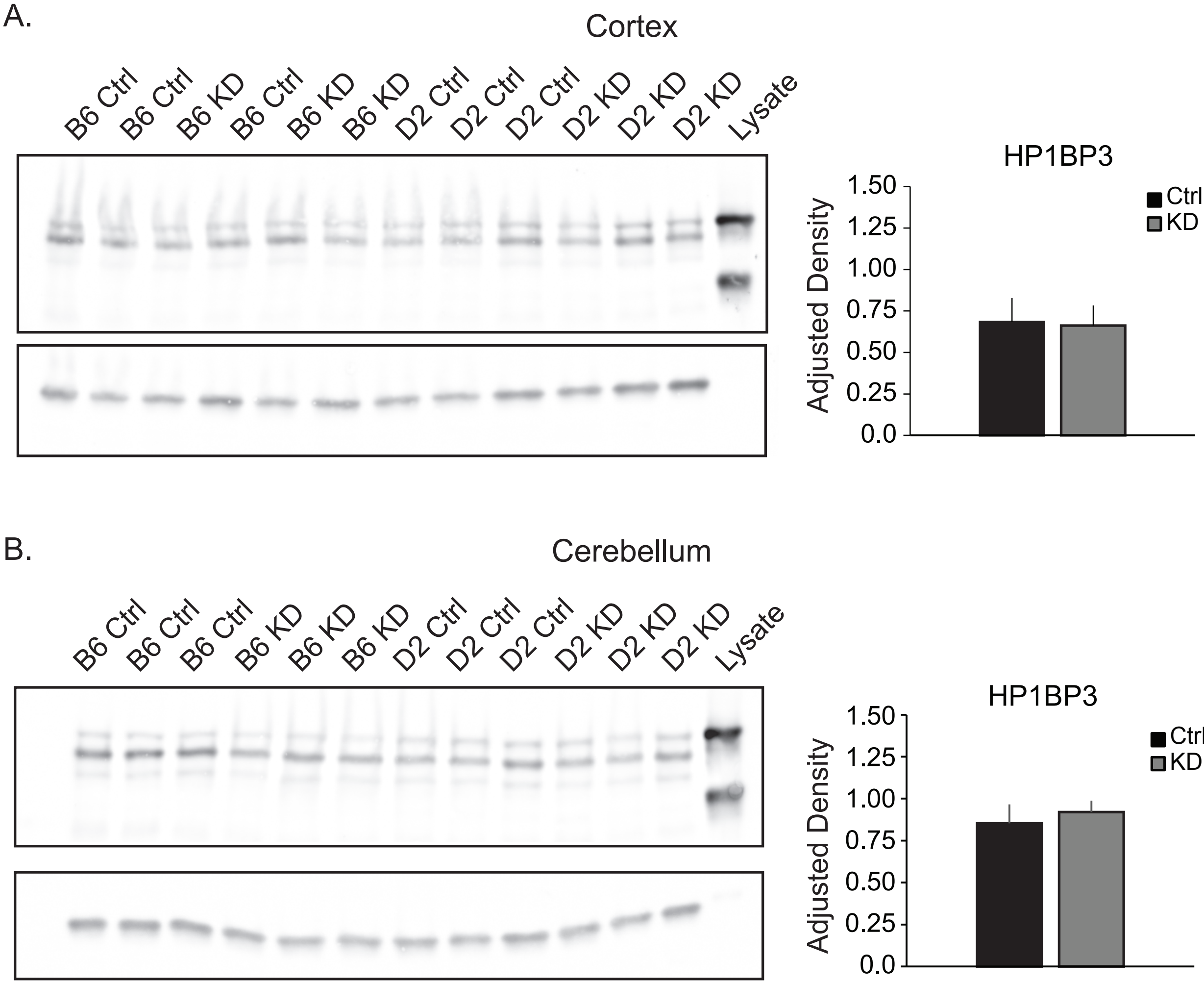

Figure S1, Related to Figure 1: Delivery of *Hp1bp3*-shRNA to the hippocampus does not effect cortical or cerebellar levels of HP1BP3. A) No effect of treatment on HP1BP3 levels in the cortex was observed on western blot (n = 3/grp, two-way ANOVA effect of strain $F(1, 8) = 4.2$, $p = 0.08$, effect of treatment $F(1,8) = 0.02$, $p = 0.9$, no interaction]. Right, band densities were adjusted for total protein loading using GAPDH levels and quantified. B) No effect of treatment on HP1BP3 levels in the cerebellum was observed [n = 3/grp, effect of strain $F(1,8) = 1.4$, $p = 0.3$, effect of treatment $F(1, 8) = 0.2$, $p = 0.6$, no interaction]. Right, band densities were adjusted for total protein loading using GAPDH levels and quantified. Lysate from human 293T cells overexpressing HP1BP3 was used as a positive control. See also Figure 1.

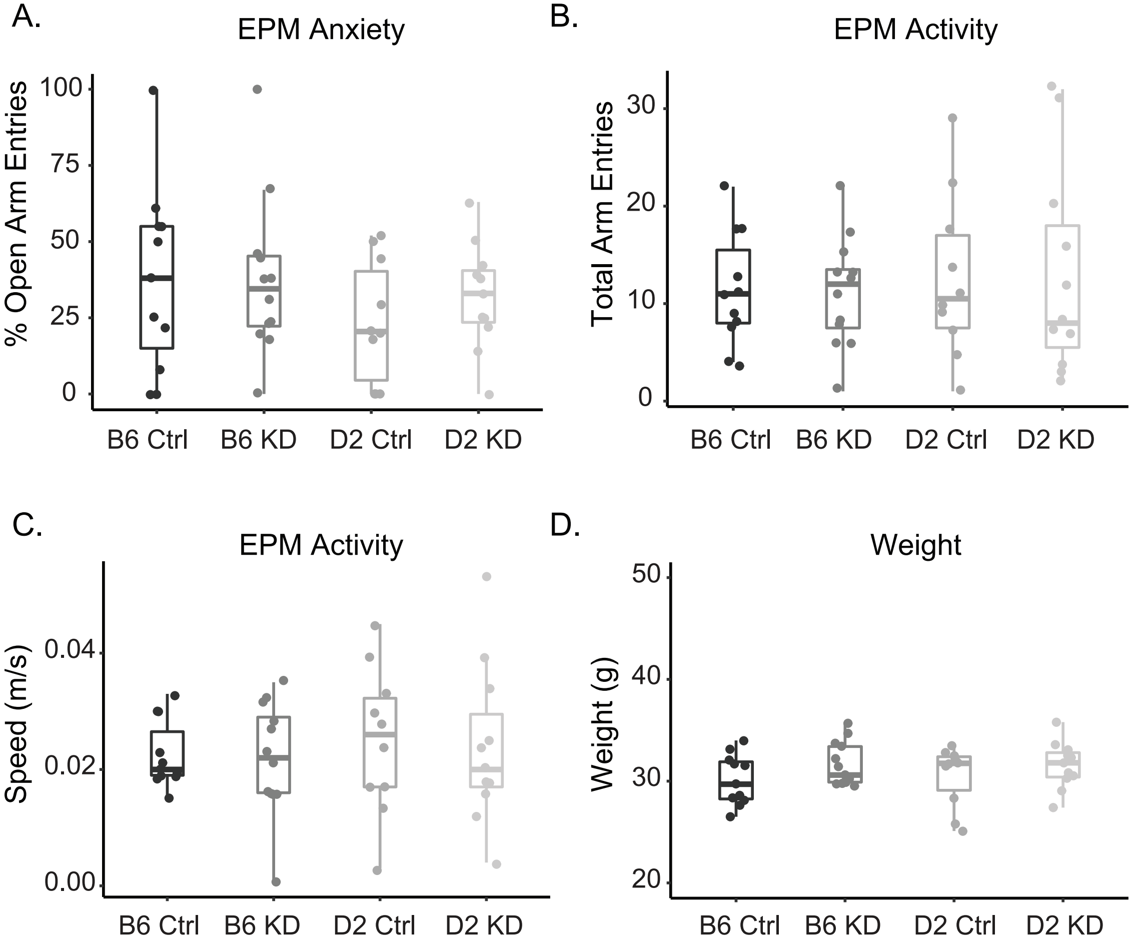

Figure S2, related to Figure 1: *Hp1bp3* KD does not have non-specific effects on anxiety, activity, or body weight. No effects of strain or treatment were observed on A) anxiety as measured by the percentage of entries made into the open arms on elevated plus maze [effect of strain $F(1, 40) = 1.8$, $p = 0.18$, effect of treatment $F(1,40) = 0.33$], B) activity as measured by total arms entered on the elevated plus maze [effect of strain $F(1, 40) = 0.39$, $p = 0.54$, effect of treatment $F(1,40) = 0.00$, $p = 0.99$], C) activity as measured by the average speed in the elevated plus maze [effect of strain $F(1, 40) = 0.46$, $p = 0.50$, effect of treatment $F(1,40) = 0.06$, $p = 0.82$], or D) body weight [effect of strain $F(1, 41) = 0.05$, $p = 0.83$, effect of treatment $F(1,41) = 3.0$, $p = 0.09$]. See also Figure 1.

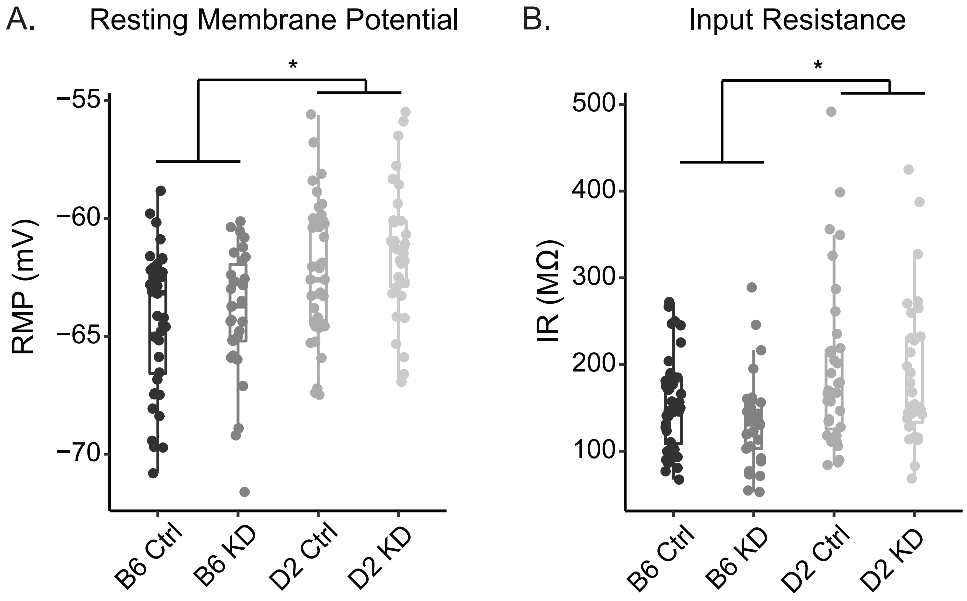

Figure S3, Related to Figure 4: *Hp1bp3* knockdown does not alter basic membrane characteristics. A) Although resting membrane potential (RMP) is different across B6 and D2 mice [two-way ANOVA, effect of strain $F(1, 136) = 22.6$, $p < 0.001$], *Hp1bp3* KD did not alter RMP [effect of treatment $F(1, 136) = 1.4$, $p = 0.2$]. B) Similarly, for input resistance (IR) although strain-specific differences are observed [two-way ANOVA, effect of strain $F(1, 131) = 12.8$, $p < 0.001$], *Hp1bp3* KD did not alter these differences [effect of treatment, $F(1, 131) = 0.9$, $p = 0.4$].

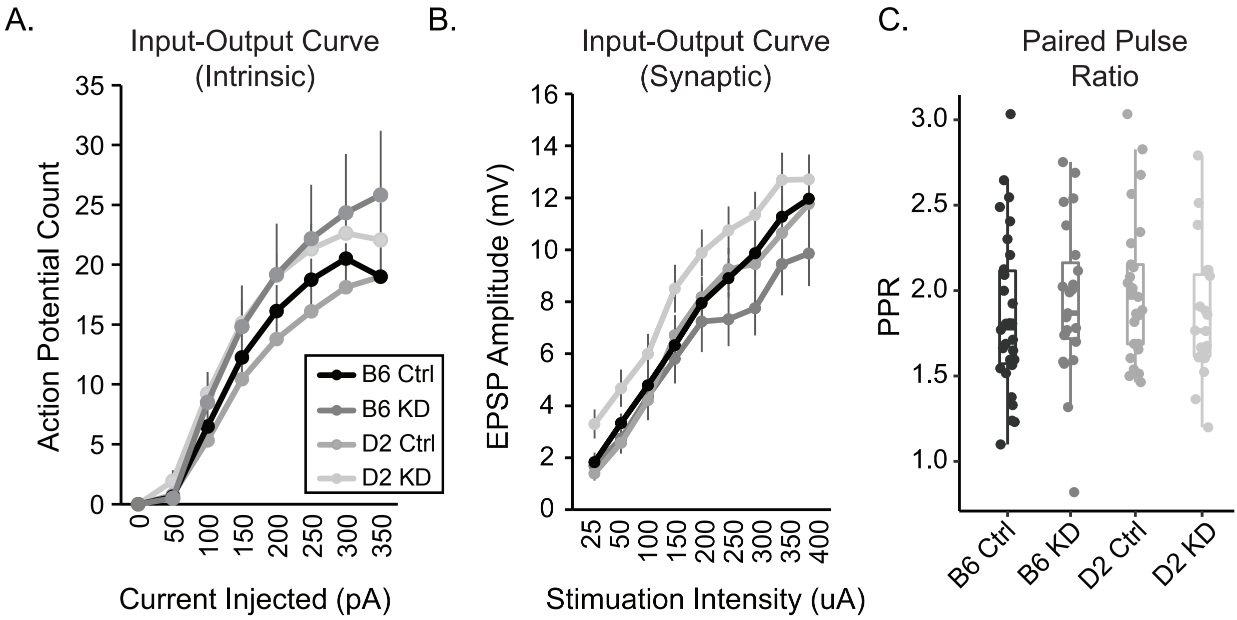

Figure S4, Related to Figure 4: *Hp1bp3* knockdown does not affect baseline firing properties or synaptic transmission. A) *Hp1bp3* KD did not change the intrinsic firing properties of hippocampal neurons, as measured by the number of action potentials fired in response to increasing 1s somatic current injections. No effect of strain [two-way repeated measures ANOVA, Greenhouse-Geisser correction $F(, 56) = 0.1$, $p = 0.8$], treatment [$F(1, 56) = 0.7$, $p = 0.4$], or interaction between strain and treatment [$F(1, 56) = 1.5$, $p = 0.2$] was detected B) The slope of evoked EPSPs increased with stimulation intensity [repeated measures ANOVA, Greenhouse-Geisser correction, effect of intensity, $F(3, 245) = 225.3$, $p < 0.001$]. No main effects of strain [$F(1, 88) = 3.3$, $p = 0.07$] or treatment [$F(1, 88) = 0.06$, $p = 0.8$], or interactions between either term and stimulation intensity [time*treatment $F(3, 245) = 1.6$, $p = 0.02$, time*strain $F(3, 245) = 1.2$, $p = 0.3$] were observed. C) Paired pulse facilitation was measured across groups. No main effects of strain [two-way ANOVA, $F(1, 94) = 0.2$, $p = 0.7$], treatment [$F(1, 94) = 0.1$, $p = 0.7$], or strain*treatment [$F(1, 94) = 1.8$, $p = 0.2$] were observed. See also figure 4.
