# Supplementary material for: Knockdown of heterochromatin protein 1 binding protein 3 recapitulates phenotypic, cellular, and molecular features of aging
Source: Aging Cell. 2018 Dec 13;18(1):e12886. doi: 10.1111/acel.12886 (PMC6351847; doi:10.1111/acel.12886)
Supplement: Supplementary file 5 [file ACEL-18-e12886-s005.pdf]

Supporting Information

Experimental Procedures

Animals

Male 3-6mo C57BL/6J and DBA/2J mice were obtained from The Jackson Laboratory (JAX #000664 and #000671, respectively). Mice were group housed and maintained on a 12 hour light/dark cycle with ad libitum access to food and water. Mouse experiments occurred at The Jackson Laboratory and the University of Tennessee Health Science Center and were conducted according to the NIH Guide for Care and Use of Laboratory Animals. All procedures and protocols were reviewed and approved by The Animal Care and Use Committee of The Jackson Laboratory and/or the UTHSC Animal Care and Use Committee where appropriate.

Design of viral vectors

Adeno-associated viral (AAV) vectors were made by Vector Biolabs (Philadelpha, PA, USA). shRNA targeting *Hp1bp3* was packaged into AAV serotype 9 vector and stored in a 1X PBS buffer containing 0.001% pluronic F-68, 0.22 mm filter sterilized. A scrambled shRNA control was also synthesized according to identical specifications. The resulting vectors were subjected to purification by 2 rounds of CsCl Density Gradient Centrifugation combined with ultracentrifugation to separate empty and full capsids.

Intrahippocampal injections

Delivery of viral vectors into the hippocampus was performed as previously described (Neuner et al., 2015). Briefly, mice were anesthetized under 4-5% inhalable isoflurane gas and once anesthetized, positioned in a stereotaxic frame and maintained at 1-2% inhalable isoflurane gas. Guide cannula and a 28 gauge microinjector attached to a Hamilton syringe and syringe pump were used to deliver 1.0 uL of virus per hemisphere at a rate of 1.0 uL/min. Microinjectors were left in place for 5 minutes following the end of injection to allow virus to diffuse adequately. Following injection, bone wax was applied to the skull and the cut was sutured using absorbable sutures. Post-surgery, topical bupivacaine was administered and mice were monitored once daily for a minimum of three days.

T-maze

Four to six weeks following viral injections, working memory was assessed on the T-maze. Mice were habituated to the testing room for three days prior to testing. Mice were placed in the start arm of the T and allowed to freely choose one of two goal arms. Upon entering an arm, mice were confined to that same arm for 30s. The mice were then placed back into the start arm and allowed to choose again. A correct alternation occurred when the mouse chose the new arm, not the arm to which it had previously been confined. Two trials per day were conducted over three days for a total of six independent trials. Results were combined to obtain a measure of average percent correct alternation.

Contextual fear conditioning

Similar to T-maze, mice were habituated to the testing room for three days prior to testing. Standard contextual fear conditioning was performed (Neuner et al., 2016; Neuner et al., 2015), with mice receiving four 0.9 mA shocks on training day. Twenty-four hours later, mice were returned to the training chamber and the percentage of time the mice spent freezing during a 10-minute test was recorded as an index of contextual fear memory.

Slice preparation and electrophysiological recordings

Hippocampal slices were prepared as described previously (Neuner et al., 2015). Briefly, mice were anesthetized with isoflurane. Brains were rapidly removed and placed in ice-cold artificial CSF (aCSF) containing (in mM): 125 NaCl, 25 Glucose, 25 NaHCO3, 2.5 KCl, 1.25 NaH2PO4, 2 CaCl2, 1 MgCl2, pH 7.5, bubbled with 95% O2/5% CO2. Acute transverse hippocampal slices (300 µm) were cut with a vibratome (Leica, VT1000S). Slices were incubated at room temperature in bubbled aCSF for 1-4 h before use. Whole-cell current clamp recordings were made in the CA1 pyramidal neurons at 30℃ under visual guidance of a video microscope (Olympus, Q-imaging digital camera) using patch pipettes with resistance of 3-5 MΩ filled with potassium gluconate based internal solution (in mM): 115 K-Gluconate, 20 KCl, 10 Na-phosphocreatine, 10 HEPES, 2 MgATP, and 0.3 NaGTP, 0.1% biocytin, pH adjust to 7.3. A multiple 700B amplifier, pClamp 10.7 software, and Digidata 1550B interface (Molecular Devices) were used to acquire data. Recordings were acquired at a 10 kHz sampling frequency and digitized at 20 kHz. Neurons were held at -67 mV (unless measuring resting membrane potential). Series resistance and capacitance were monitored and compensated throughout recordings. Neurons with >40 mΩ series resistance were excluded. The post-burst afterhyperpolarization (AHP) was triggered using 25 brief (2

ms) somatic current injections (1 nA) at 50 Hz. Membrane properties including AHP were assessed as previously described (Kaczorowski et al., 2011).

Western blot

Whole hippocampal lysates were prepared as previously described (Neuner et al., 2016). Protein concentration was determined using a NanoDrop Spectrophotometer 2000 and 20 μg total protein was loaded onto a BioRad TGX Mini Protean gel. Proteins were separated by electrophoresis and transferred to a nitrocellulose membrane using the Trans-Blot Turbo system (BioRad). Membranes were blocked in 5% non-fat milk in PBST and primary antibodies for HP1BP3 (gifted by Drs. Benjamin Garfinkel and Joseph Orly) and GAPDH (Fitzgerald Industries #10R-G109A) were incubated at 4C overnight. Fluorescently labeled secondary antibodies were incubated for 1 hour at RT and bands were visualized using an Azure Biosystems gel imager. Observed double band staining is typical expression pattern for HP1BP3 (Garfinkel et al., 2015) and overlaps with positive control HP1BP3 overexpression lysate from human 293T cells (Abnova #H00050809-T02), which was used as a positive control. As such, both bands were included in our analysis. Band densities were measured using LI-COR Image Studio Lite (LI-COR Biosciences). Densities were first normalized to an internal B6 control, and then total HP1BP3 was adjusted for total protein loaded using GAPDH. Adjusted densities are plotted in Figure 1B and Figure S1.

RNA sequencing

Total RNA was prepared from whole hippocampal homogenates using the Qiagen miRNeasy Mini kit (#217084). RNA quality was assessed using a BioAnalyzer and only samples with RNA Integrity Number (RIN) greater than 8.0 were included. Samples were submitted to The Jackson Laboratory Genome Technologies department, where mRNA libraries were prepared using the KAPA Biosystems mRNA Hyper Prep Kit while miRNA libraries were prepared using the Illumina TruSeq Small RNA Library Prep Kit. The Illumina HiSeq 2500 was used to sequence 75 paired-end reads, at a depth of 30 million reads for mRNA and 10 million reads for miRNA. Two pairs of mRNA/miRNA samples given identical barcodes during the initial sequencing, so all samples were re-sequenced and where available, fastq files from both runs were concatenated, providing expanded sequencing depth for 10/12 samples. Poor quality mRNA reads were filtered, trimmed, and aligned to either the C57BL/6J or DBA/2J genomes where appropriate using rsem v1.2.12, followed by bowtie2 v2.2.0 for expression estimate of trimmed reads (Li and Dewey, 2011). For miRNA, trimmed reads were aligned to the mouse miRNA-precursor miRBase reference (miRBase release 21) using bowtie v1.0.0 (Griffiths-

Jones, 2006; Kozomara and Griffiths-Jones, 2014). Following alignment miRNA counts were extracted using featureCounts function from the subread package v1.5.2 (Liao et al., 2013).

Differential expression analysis

For both RNA types, expected read counts were used to perform differential expression analysis using DESeq2 according to established protocols (Love et al., 2014). miRNAs of interest were checked for single nucleotide polymorphisms (SNPs) using the Sanger Mouse Genomes database (Keane et al., 2011) to ensure observed differences were not due to strain-specific differences in miRNA sequence. For graphing of mRNA and miRNA differences across groups, normalized counts were extracted from DESeq2 using the counts function. miRNA counts were further log transformed using the rlog function.

Gene set enrichment analyses

For gene set enrichment analysis (GSEA), all genes nominally significantly differentially expressed relative to treatment (*Hp1bp3* KD vs Ctrl unadjusted p-value < 0.05) were sorted into a ranked list by $\log_2$ fold change, with the most positively differentially expressed genes at the top of the list. This pre-ranked gene list was uploaded into the GSEA desktop software (Subramanian et al., 2005). GSEA was used according to established procedures to identify significant overlap between genes positively or negatively changed by treatment and gene sets in the Molecular Signatures Database 3.0 (Liberzon et al., 2011). The same list of differentially expressed genes was uploaded into Ingenuity Pathway Analysis [IPA, Qiagen, (Kramer et al., 2014)] and the Core Analysis function was utilized to identify upstream miRNA regulators of observed mRNA changes. Specifically, we filtered results to include references from the Ingenuity Knowledge Base, included both direct and indirect connections, and considered only molecules and/or relationships which had been experimentally observed or had high predictive value. We then filtered the putative upstream regulators list to only include regulators of molecule type equal to "miRNA" or "mature miRNA". Finally, to perform simple over-representation enrichment analysis on small gene sets (e.g. miRNA target genes) as opposed to searching specifically for enrichment among up or down-regulated genes (as in GSEA), we utilized WebGestalt (Wang et al., 2013) to identify significantly enriched GO Biological Process terms.

Statistical analysis and data availability

All experiments and analysis were conducted with experimenters blind to treatment group. All statistics were performed using SPSS (IBM) or R and tests used included independent t-tests, two-way ANOVA, and two-way repeated measures ANOVA. Our LTP data used in the two-way repeated measures ANOVA violated Mauchly's test of sphericity ($p < 0.05$), so Greenhouse-Geisser corrected p-values were used for analysis. Unless otherwise stated, data values here are given as mean ± standard error. All raw and processed data has been deposited to Gene Expression Omnibus (GEO) and is available as SuperSeries GSE119321. Specifically, the mRNA sequencing data is available as GSE119318 and the miRNA sequencing data is available as GSE119319. All software packages used in R for data analysis are freely available.

References Cited

Garfinkel, B.P., Melamed-Book, N., Anuka, E., Bustin, M., and Orly, J. (2015). HP1BP3 is a novel histone H1 related protein with essential roles in viability and growth. Nucleic Acids Res *43*, 2074-2090.

Griffiths-Jones, S. (2006). miRBase: the microRNA sequence database. Methods Mol Biol *342*, 129-138.

Kaczorowski, C.C., Sametsky, E., Shah, S., Vassar, R., and Disterhoft, J.F. (2011). Mechanisms underlying basal and learning-related intrinsic excitability in a mouse model of Alzheimer's disease. Neurobiology of aging *32*, 1452-1465.

Keane, T.M., Goodstadt, L., Danecek, P., White, M.A., Wong, K., Yalcin, B., Heger, A., Agam, A., Slater, G., Goodson, M., *et al.* (2011). Mouse genomic variation and its effect on phenotypes and gene regulation. Nature *477*, 289-294.

Kozomara, A., and Griffiths-Jones, S. (2014). miRBase: annotating high confidence microRNAs using deep sequencing data. Nucleic Acids Res *42*, D68-73.

Kramer, A., Green, J., Pollard, J., Jr., and Tugendreich, S. (2014). Causal analysis approaches in Ingenuity Pathway Analysis. Bioinformatics *30*, 523-530.

Li, B., and Dewey, C.N. (2011). RSEM: accurate transcript quantification from RNA-Seq data with or without a reference genome. BMC Bioinformatics *12*, 323.

Liao, Y., Smyth, G.K., and Shi, W. (2013). The Subread aligner: fast, accurate and scalable read mapping by seed-and-vote. Nucleic Acids Res *41*, e108.

Liberzon, A., Subramanian, A., Pinchback, R., Thorvaldsdottir, H., Tamayo, P., and Mesirov, J.P. (2011). Molecular signatures database (MSigDB) 3.0. Bioinformatics *27*, 1739-1740.

Love, M.I., Huber, W., and Anders, S. (2014). Moderated estimation of fold change and dispersion for RNA-seq data with DESeq2. Genome biology *15*, 550.

Neuner, S.M., Garfinkel, B.P., Wilmott, L.A., Ignatowska-Jankowska, B.M., Citri, A., Orly, J., Lu, L., Overall, R.W., Mulligan, M.K., Kempermann, G., *et al.* (2016). Systems genetics identifies Hp1bp3 as a novel modulator of cognitive aging. Neurobiology of aging *46*, 58-67.

Neuner, S.M., Wilmott, L.A., Hope, K.A., Hoffmann, B., Chong, J.A., Abramowitz, J., Birnbaumer, L., O'Connell, K.M., Tryba, A.K., Greene, A.S., *et al.* (2015). TRPC3 channels critically regulate hippocampal excitability and contextual fear memory. Behavioural brain research *281*, 69-77.

Subramanian, A., Tamayo, P., Mootha, V.K., Mukherjee, S., Ebert, B.L., Gillette, M.A., Paulovich, A., Pomeroy, S.L., Golub, T.R., Lander, E.S., *et al.* (2005). Gene set enrichment analysis: a knowledge-based approach for interpreting genome-wide expression profiles. Proceedings of the National Academy of Sciences of the United States of America *102*, 15545-15550.

Wang, J., Duncan, D., Shi, Z., and Zhang, B. (2013). WEB-based GEne SeT AnaLysis Toolkit (WebGestalt): update 2013. Nucleic acids research *41*, W77-83.
