# Supplementary material for: Knockdown of heterochromatin protein 1 binding protein 3 recapitulates phenotypic, cellular, and molecular features of aging
Source: Aging Cell. 2018 Dec 13;18(1):e12886. doi: 10.1111/acel.12886 (PMC6351847; doi:10.1111/acel.12886)
Supplement: Supplementary file 6 [file ACEL-18-e12886-s006.pdf]

Data S1: Experimental Procedures

Table S1, Related to Figure 2: List of genes differentially expressed relative to strain, treatment, or that display a significant interaction between strain and treatment. See also Figure 2.
*See Excel spreadsheet.

Table S2, Related to Figure 6: DESeq2 results from analysis aimed at identifying differentially expressed miRNAs relative to strain, treatment, or which display an interaction between strain and treatment. See also Figure 6.
*See Excel spreadsheet

Table S3, Related to Figure 6: List of miRNAs identified by Ingenuity Pathway Analysis as putative upstream regulators of observed mRNA changes after *Hp1bp3* KD. See also Figure 6.
*See Excel spreadsheet
